# Pulsed electric field effect on acrylamide reduction and quality attributes of continuous-style Lamoka potato chips

**DOI:** 10.1016/j.heliyon.2024.e31790

**Published:** 2024-05-29

**Authors:** Priscila Santiago-Mora, Mark Skinner, Alyssa Hendricks, Tauras Rimkus, Brian Meyer, Jim Gratzek, Shin Pu, Luke Woodbury, Laura Bond, Owen McDougal

**Affiliations:** a Department of Chemistry and Biochemistry, Food and Dairy Innovation Center, Boise State University, 1910 W University Dr, Boise, ID, 83725, USA; b Food Physics, 8512 W Elisa St, Boise, ID, 83709, USA; c Biomolecular Research Center, Boise State University, 1910 W University Dr, Boise, ID, 83725, USA

**Keywords:** Potato chips, Pulsed electric field, Acrylamide, Lamoka, Continuous frying

## Abstract

Potato chips are a popular snack, well-liked because of their texture-flavor combination. Potato chips are made by frying slices of potato in vegetable oil to achieve a crispy texture. Frying potato slices initiates the Maillard reaction, resulting in chemical changes that enhance taste, color, and texture, but also undesired acrylamides, which are suspected carcinogens. The application of pulsed electric field (PEF) technology is commonly used in French fry processing operations to prolong cutting blade sharpness and reduce waste, energy consumption, and water usage. Despite these attributes, PEF systems have not yet gained widespread adoption by potato chip producers. In the current study, Lamoka potatoes were PEF-treated prior to continuous frying into potato chips. The effect of specific energy at 0.75 kJ/kg (Low-PEF) and 1.5 kJ/kg (High-PEF) and electric field strength of 1 kV/cm, frequency of 24 kV, and pulse width of 6 μs versus untreated (control) samples was studied, then batches of 250 g of slices were fried at 170 °C or 185 °C for two frying times to obtain potato chips with acrylamide levels below the California Proposition 65 limit (275 ng/g). The Lamoka potato chip product quality metrics that were assessed include moisture, fat, reducing sugars, asparagine, acrylamide, chip color, and texture. PEF treatment of Lamoka potatoes resulted in chips fried in 10 % less time, lower oil content by 8 %, and a decrease of reducing sugars by 19.2 %, asparagine by 42.0 %, and acrylamide by 28.9 %. The PEF fried chips were lighter in color but maintained textural attributes compared to continuous frying cooking. The process of frying potato slices at 170 °C for 150 s with High-PEF yielded potato chips with acrylamide content below the California Proposition 65 limit; which speaks to the health implications for consumers and the quality and safety of these chips.

## Highlights

• Frying time for PEF-treated Lamoka potato slices can be reduced by 10 %.
• PEF reduces acrylamide precursors, reducing sugars by 19 %, and asparagine by 42 %.
• Chips from PEF-treated potatoes have less oil content by 8 %, and acrylamide by 29 %.
• PEF-treated chips are lighter in color with texture comparable to untreated chips.
• PEF-treated potatoes produce chips sustainably and with better nutritional value.

## Graphical abstract

**Figure:**
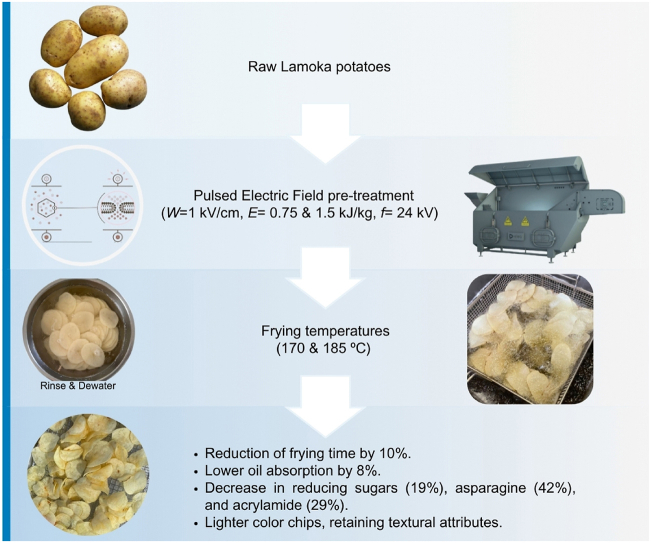

**Industrial relevance text**

The findings of this study support the use of PEF for continuous frying potato chip processing. The benefit of PEF pre-treated chips is that they can be produced more economically and sustainably, with better nutritional value for consumers.

## 1 Introduction

Potato chips are popular with consumers because of their texture-flavor combination. Potato chips are generally made by frying a thin slice of potato in vegetable oil to achieve a crispy texture. Frying potato slices in oil initiates the Maillard reaction, which produces sensory molecules that enhance taste, color, and texture, all of which are desirable for potato chips. One undesired compound resulting from the Maillard reaction is acrylamide, this chemical has been found to produce cancer in lab animals and has negative effects on reproductive health [1].

The present study aimed to examine the effect of PEF pre-treatment on continuous fry potato chips, to reduce acrylamide as well as describe the impact of introducing this pre-treatment on the quality and sensory attributes of the chips. This is the first study focused on Lamoka potatoes, the most common chipper variety in the United States. Our investigation sought to evaluate whether PEF-treatment of Lamoka potatoes led to viability of the production of chips, with improved nutritional value for consumers.

The specific varieties of potatoes suitable for chips are known as “chippers”. Chipping varieties of potatoes grown in the United States include Atlantic, Beacon Chipper, Huron Chipper, Mackinaw, Manistee, Hodag, Snowden, Lamoka, and Lady Liberty [2]. Of these varieties, Lamoka is the most common chipper in the United States [3] due to its size, shape, and reducing sugar content. Lamoka potatoes are round and medium-sized, with a diameter of about 7 cm [4], which makes for efficient peeling and optimal slice size to fit into one's mouth for favorable consumer satisfaction. Lamoka potatoes also have low glucose content, on the order of 0.25 mg/g of tuber, which yields a light-colored chip when cooked [5].

### 1.1 Potato chip production process

Potato chip processing involves frying potato slices (1.2–2.0 mm thick) [6], in vegetable oil at temperatures up to 190 °C to achieve desirable quality attributes including texture, color, and oil content in the chip [7]. During frying, the water content in potato slices is reduced from 75-80 % to 1.0–2.5 % [6], resulting in a finished product of oily and dry consistency. It is the high temperature and oil infiltration into the potato slices during cooking that induces the major structural transformation resulting in the flavor, color, and texture combination that is appealing to consumers [8].

The two common frying processes for potato chips are continuous and kettle, a. k.a., batch. In the continuous fry method, oil in the fryer is preheated to a temperature above 175 °C. Potatoes are washed, peeled, sliced to a thickness of 1.3 mm, and the slices are washed to remove starch from their surface before frying. When the potato slices are added to the oil, the temperature generally drops on the order of 15 °C throughout the cooking time. While in the oil, the chips are moved from one side of the fryer to the other, with constant stirring by paddles, hence, the method is termed “continuous frying”. From start to finish, the oil temperature drops, and the fry time is generally completed within 180 s to achieve a final moisture content in the chips between 1.3 and 1.5 %. Kettle chips are made by batch frying. Potatoes are washed, peeled, sliced to a thickness of 1.6 mm, and the slices are not washed prior to frying. Kettle chips have characteristic thicker slices with more “crunch” due to the starch left on their surface. The oil temperature for batch frying is generally lower than that used for the continuous frying process. An oil temperature of 140 °C serves as an initial point for potato slice submersion, which causes a drop-in oil temperature, followed by heating to increase the oil temperature to a final state of around 148 °C. Since kettle chips are thicker and the oil temperature is lower, the cooking time can be as long as 540 s to achieve a final moisture content of 1.8–2.0 %. The details associated with continuous and batch frying of potato chips are provided in Table 1.

**Table 1:** Potato chips processed by continuous or batch frying methods.

Frying Style	Continuous	Batch
**Slice thickness**	1.3 mm	1.6 mm
**Post slicing**	Starch removed by washing	Starch not removed
**Inlet fry temperature**	175 °C	140 °C
**Outlet fry temperature**	160 °C	148 °C
**Fry time**	180 s	540 s
**Moisture content**	1.3–1.5 %	1.8–2.0 %

### 1.2 Quality attributes of potato chips

The attributes of potato chips that make them popular snack foods include their moisture and fat content, color, and texture. The moisture content for continuous fry chips is generally between 1.3 and 1.5 %, whereas kettle chips are between 1.8 and 2.0 %. Chips rarely have moisture levels above 2.5 %, because, at higher moisture, the chips have a shorter shelf life and bland color that is less than desirable [5]. The moisture content of potato slices drops from nearly 80 % to around 2.0 % during frying. As water is removed from the potato during cooking, oil begins to infiltrate the chip. The amount of oil in the finished product can range from 28 % in the continuous fry process to as much as 48 % in kettle chips [5]. The texture, flavor, and mouth feel of the finished product are largely dependent on the oil used during frying. The most common oils for potato chip frying are corn, sunflower (mid-oleic and high-oleic varieties), canola, high-oleic safflower, and cottonseed oils [9]. The characteristics of these oils that make them good choices for chip frying are their clear color, bland flavor, and low free of fatty acid content (≤0.2 %).

The color of potato chips is critically important to consumer perception and satisfaction. As mentioned above, the color of finished chips may be influenced by the selection of fryer oil, and also whether the oil has been filtered frequently and stored appropriately to prevent degradation. To assess chip quality, color accounts for 30 % of the final product evaluation standard [5]. In addition to the oil, potato chip color is dependent on the amount of reducing sugars and asparagine present in the raw potato, and the frying temperature and cooking time [10]. The impact on color development through the Maillard reaction and during the frying of potato chips is shown to be cultivar-dependent [11].

For potato chips, texture is very important. The optimal characteristics for potato chips are to be firm, crispy, and crunchy. Firmness is measured as the maximum force (N) required to break the chips [12]. Firmness depends on the degree of starch gelatinization and changes in the cell walls’ structure [13]. The term crispness is associated with mechanical failure, and crunchiness is associated with sound bursts during chewing. Force-deformation test systems give a qualitative representation of the overall jaggedness of the original curve directly related to crispiness (n), and crunchiness (N∙s) in potato chips [14].

### 1.3 Acrylamide – a challenge in potato chip production

The moisture and fat content, color, and texture of potato chips are significantly influenced during frying due to heat catalyzing the Maillard reaction of asparagine and reducing sugars to produce the brown melanoidins for color, and sensory molecules for taste, [15,16]. The Maillard reaction is one of the most important chemical reactions in food processing [17], and the extent to which its sensory molecules are produced is dependent on water activity, asparagine and reducing sugar levels, fryer oil temperature and cook time [18]. Not only does the Maillard reaction produce desirable sensory molecules, but it also yields acrylamide, a possible carcinogen in humans that has led to regulation in the state of California and in the European Union due to public safety concerns [19].

Acrylamide is classified by the International Agency for Research on Cancer & World Health Organization, (1994) as a Group 2A carcinogen “probably concerning to humans” due to its carcinogenic implications in rats [20]. Acrylamide is on the California Proposition 65 list because it can cause cancer, birth defects, or other reproductive harm [21]. Acrylamide in food is generally well below levels currently believed to cause harmful effects in humans. California Proposition 65 requires potato chip producers to include a warning label on the package if acrylamide exceeds 275 ng/g [1], whereas the European Commission is 750 ng/g of acrylamide for potato chips [22]. As of today, no direct correlation between acrylamide exposure and human cancer has been established.

While acrylamide is present in all carbohydrate-rich foods processed at high temperatures, potato chips are among the highest contributors of the toxin to the human diet in developed countries [23]. Since the discovery of acrylamide in heat-processed foods [24], significant efforts have been undertaken by researchers and food processors around the world to find industry-suitable solutions to prevent or decrease acrylamide levels in foods [22,[25], [26], [27]].

Fried chips are highly susceptible to acrylamide formation due to the high surface volume ratio, glucose, fructose, and asparagine content as well as their exposure to temperatures above 120 °C [28]. Acrylamide values for potato chips range from 108 to 3444 μg/kg [29]. Acrylamide is not present in raw potatoes, and its formation is cultivar-specific, due to the content of reducing sugars and asparagine in the tuber, and dependent on frying time and temperature [30]. Chipper potato varieties like the Lamoka are bred to have low levels of reducing sugars and asparagine.

#### 1.3.1 Acrylamide mitigation strategies

Eight strategies have been studied to reduce acrylamide formation in fried potato products [31] that include the use of potato breeding varieties that produce low levels of asparagine and reducing sugars, application of enzymes, acids, and salts, alteration to slice thickness, fryer temperature, and fry time, potato peeling and blanching, yeast, and sorting by color. Table 2 provides an overview of the advantages and disadvantages of each of these acrylamide mitigation strategies, along with a reference to the original scientific study that reported the result.

**Table 2:** Strategies to reduce acrylamide formation in potato products.

Strategy	Advantage	Disadvantage	Reference
Reducing sugar content and the fructose/asparagine (Asn) ratio <2 in the potato variety, ensuring tubers are mature at the time of harvest (immature tubers tend to have higher reducing sugar levels), controlling storage temperature to 6 °C and 95 % relative humidity.	The concentration of reducing sugars is generally regarded as a good indicator of the relative acrylamide-forming potential of different batches of tubers of the same potato variety.	Breeding opportunities with lower reducing sugars and/or less cold sweetening effect.	[32]
Selection of crop varieties based on free Asn/total free amino acid ratio	The ratio of Asn/total free amino acids is of great significance to the formation of acrylamide in potatoes.	The impact of fertilizers may have an effect on amino acid ratios in potatoes. Nitrogen and sulfur fertilization may alter the ratio of free Asn/total free amino acids in a tuber. The effects of fertilization are variety-specific, and so far, no optimum amino acid ratio in potatoes has been established.	[33]
The use of disodium pyrophosphate, organic acids, divalent cations, and asparaginase to reduce acrylamide formation.	Some potato products like wet doughs may use these additives in their formulations.	These additives reduce acrylamide in an optimized laboratory environment, but they are ineffective in pilot and industrial settings, resulting in high costs and negative sensory modifications.	[8,27,28,34]
Slice thickness, a thin-cut potato chip product theoretically requires less thermal input for the same fry time to reach the same moisture endpoint, which lowers acrylamide formation.	A thin-cut potato chip would require less thermal input for the same fry time to reach the same moisture endpoint, and so offers lower acrylamide forming potential.	Slice thickness is, however, an important product characteristic for sliced potato chips and is difficult to address without fundamentally altering the finished product.	[35]
Modifying the thermal input and moisture. The fryer exit temperature should not be higher than 168 °C. Moisture content has a strong influence on the activation energy of browning and acrylamide formation. The end phase of the frying process is critical and must be carefully controlled at lower product temperatures to optimize color development and minimize acrylamide formation.	Thermal input controls acrylamide formation in the finished product.	Vacuum frying offers an alternate thermal input control system; however, this technology has limited throughput capacity and may not deliver desired product attributes given the lack of Maillard components formed, additionally, this frying leads to much harder products.	[36]
Peeling potatoes before frying.	Reducing sugars are higher in the skin layer than in the potato flesh.	The impact of peeling on overall acrylamide levels is highly dependent on the potato variety and season.	[37]
Blanching may be an option in some potato products like potato sticks.	Blanching can be used to remove sugars to reduce acrylamide levels.	Blanching has a negative impact on the texture of the final product.	[27]
Lactic acid fermentation using acrylamide-reducing yeast has been proposed to reduce the availability of Asn prior to and/or during processing.	Fermentation reduces levels of key reactants for the formation of acrylamide and lowers pH.	For sliced potato chips the yeasts cannot penetrate the potato slice sufficiently to act upon the asparagine^1^, besides, extended pre-treatment of the slices with the yeast could disintegrate the slice structure.	[38]
In-line color sorting: darker color products are more likely to have come from tubers that are higher in reducing sugars, increasing the acrylamide level by up to 25–50 %.	In-line color sorting can be an effective measure to remove darker products that may have a higher acrylamide level.	Color alone, cannot be used as a substitute for analytical acrylamide quantification.	[39]

Table 2 shows the common ways that have been attempted to lower acrylamide levels in fried potato products. The strategies suggested by FoodDrinkEurope Toolbox (2019) mentioned the patented Pulsed Electric Field pretreatment as an alternative to reduce acrylamide in potato-based products by allowing Asn and reducing sugars to be washed out of the potato. The focus of the current work is on the use of pulsed electric field technology to achieve acrylamide reduction in potato chip results commensurate with or superior to the mitigation strategies that are typically utilized in the industry.

#### 1.3.2 Pulsed electric field

PEF technology generates repetitive electrical pulses of high voltage for a short time (nanoseconds or milliseconds) that when applied to food material placed between two conducting electrodes, promotes the loss of the semi-permeability of cell membranes in biological tissues, favoring mass transfer phenomena [40]. PEF treatment may be tuned for mild oxidative stimulation of metabolic function or extreme disintegration of the cell membranes, depending on treatment intensity [17]. PEF processing parameters include electric strength (*E*), pulse frequency (*f*), pulse number (*N*), pulse shape, specific energy input (*W*), pulse width (τ), and pulse duration (*t*) [41].

The application of PEF for potato pre-treatments above 0.2–1.0 kV/cm of field strength is well known to enhance mass transfer from plant tissues, increasing the cell membrane permeability [42]. Following this principle, Jaeger et al. (2010) assert that PEF treatment of raw potatoes may assist and increase the release of simple sugars and free amino acids that represent the main substrates for the Maillard reaction, consequently lowering the formation of acrylamide. Genovese et al. (2019) described the reduction of acrylamide precursors in raw potatoes of the Lady Claire variety by the application of PEF pre-treatment followed by 5-min water dipping and fried in high-oleic sunflower oil at 175 °C for 3 min, which led to a reduction in acrylamide content by 30 % in potato chips compared to an untreated sample. Janositz et al., 2011 reported a one-third reduction of fructose content and a near-half reduction of glucose in the release of reducing sugars in potato slices from Karlena and Satuna varieties, after PEF treatments of 1.5 kV/cm and 20 pulses.

In addition to the application of PEF-induced cell electroporation for the removal of reducing sugars and free amino acids, industrial application of PEF to potatoes generally occurs before cutting to soften the potato tuber tissues and improve their processing textural quality [43,44]. PEF-treated potatoes exhibit higher retention of starch [43], lower uptake of fry oil [43,44] and a more uniform color during frying [44]. The PEF-induced electroporation of potato cell membranes by pre-treatment enhances diffusion properties during frying that accelerate the thermal process [45], resulting in shorter frying times and a reduction in acrylamide formation during the deep-frying step [28]. Over 100 machines are used in the potato industry around the world, both for French fries and potato chips [46].

## 2 Materials and methods

### 2.1 Chemicals

Hexanes (98.5 % purity, 5 mL), formic acid (98 % purity), methanol (99.9 % purity), acetonitrile (99.9 % purity, 10 mL), petroleum ether (99.9 % purity, 100 mL), ethanol (33 %, 10 mL), and phenol: chloroform: isoamyl alcohol standard (25:24:1, 1 mL) were sourced from Fisher Scientific (Waltham, USA). Acrylamide ^13^C_3_ (99 %, 500 μL) and unlabeled acrylamide were sourced from Cambridge Isotope Laboratories (Tewksbury, MA); both stored at −5 °C, protected from light. QuEChERS (Quick, Easy, Cheap, Effective, Rugged, and Safe) extraction kit and AOAC Dispersive SPE kit were sourced from Agilent (Santa Clara, USA).

### 2.2 Raw materials

Fresh potatoes (*Solanum tuberosum*, Lamoka variety) harvested in September during peak conditions, were donated by Pleasant Valley Potato, Inc. Aberdeen, ID USA, and stored at 8 °C for use within one week after being received. No special selection postharvest was used other than the rejection of tubers with visual defects. Canola oil (Harvest Value, Boise, USA) was used in all frying runs.

### 2.3 Sample processing

Potatoes were washed and peeled for 2 min using a mechanical peeler (Omcan, Brazil). PEF treatment was carried out (or not for control) with rectangular pulses of high energy using an Elea PEF Advantage ™ Belt One system (Quakenbrück, Germany), where potatoes are being transported with a conveyor belt at a speed of 0.14 m/s into a water bath with a conductivity of 800 μS/cm where the electrodes are placed at 24 cm distance. The effect of specific energy (*W*) at 0.75 kJ/kg (Low-PEF) and 1.5 kJ/kg (High-PEF) and fixed electric field strength (*E*) of 1 kV/cm, frequency (f) of 24 kV, and pulse width (τ) of 6 μs versus untreated (control) samples were studied. The specific energy is the total energy applied per mass unit of the product and can vary between 0.5 kJ/kg – 2.0 kJ/kg for potatoes. The electric field strength is the intensity of the field between two electrodes and is important for the electroporation of the cells and is dependent on the foodstuff [47]; remaining constant at 1 kV/cm for the trials. The pulse width is defined as the time when the peak field is maintained [48], many short pulses were used to avoid an increase in temperature. It was observed that there were no temperature changes in the potatoes after PEF treatments. The combination of PEF parameters used in this study was selected based on literature-reported preliminary studies [47,49]. Untreated and PEF-treated potatoes were sliced to a thickness of 1.4 mm with an Urschel CC slicer (Chesterton, USA); to observe uniformity of the thickness, the center and edges of the potato slices were measured with a digital thickness gauge (Mitutoyo H-2781, Japan). The sliced samples were subsequently submerged in 5 L of room temperature (20 ± 2 °C) tap water for 1 min with constant manual agitation. Tap water at cold, hot, and room temperatures was tested previously to identify a significant effect on the release of reducing sugars, however, no effect was noted (data not included). The submerged slices were strained and pat-dried with paper towels. Samples for analysis were collected after peeling, and dewatering.

### 2.4 Frying conditions

**Fig. 1:**
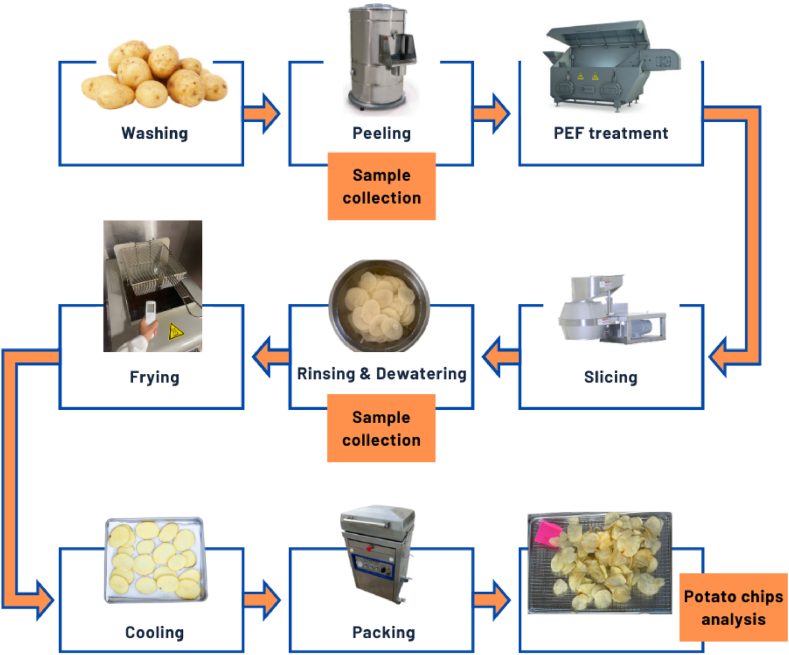
Flowchart of experimental frying of samples.

Fig. 1 illustrates a flowchart of the frying experiments performed for each treatment condition. Three hundred and 50 g of potato slices constituted a batch of chips for each fryer trial. Frying conditions were intended to replicate the continuous style processing by having constant stirring in the fryer and observing a temperature drop of 15 °C as expected in this frying curve. Replicates consisted of three batches of chips that were fried under the same conditions. Our frying times and experiments were controlled as a function of the final moisture reached in the potato chips after PEF pre-treatment. The experimental conditions of fryer oil temperature were 170 °C and 185 °C, fry time of 135 or 150 for the lower temperature and 150 and 180 s for the high temperature, as well as PEF treatment conditions that included an untreated control, a Low-PEF of 0.75 kJ/kg and a High-PEF of 1.5 kJ/kg (Table 3).

**Table 3:** Experimental conditions of fryer oil temperature, cook time, and PEF-specific energy. These parameters were selected to demonstrate representative fryer temperatures used in the potato chip industry. The parameters selected demonstrated a high and low frying temperature each with a short and long frying time.

Frying temperature	Frying time	PEF treatment
170 °C	150 s	Control
		Low-PEF (0.75 kJ/kg)
		High-PEF (1.5 kJ/kg)
170 °C	180 s	Control
		Low-PEF (0.75 kJ/kg)
		High-PEF (1.5 kJ/kg)
185 °C	135 s	Control
		Low-PEF (0.75 kJ/kg)
		High-PEF (1.5 kJ/kg)
185 °C	150 s	Control
		Low-PEF (0.75 kJ/kg)
		High-PEF (1.5 kJ/kg)

The variable conditions were determined after five repeated frying curves, where the variable frying time was adjusted based on PEF treatment and after evaluating the final moisture content (≤2.5%) and the physical appearance of the potato chips. All frying was performed in a custom-made deep-fat oil electrical 11 kW fryer ELEA (Quakenbrück, Germany) with a thermocouple located in the center of the fryer that allows for instant monitoring of temperature fluctuations during frying; any temperature fluctuation above 2 °C automatically restarts the seven heating elements to compensate for the heat loss. Sampling intervals were determined experimentally. When the oil reached the targeted temperature, the frying basket was immersed in the hot oil. Fried slices were drained over a wire screen for 5 min and allowed to cool to room temperature before moisture content was determined and the remaining samples were packed in sealed bags with nitrogen for further analysis. The fryer was filled with 15 L of oil and preheated for 1 h before frying.

### 2.5 Analytical determinations

#### 2.5.1 Moisture content

Moisture content was determined gravimetrically using the AOAC #984.25 method and by moisture analysis (Ohaus, MB120, Parsippany, NJ, USA) to compare precision. The gravimetric method is based on the mass loss after the sample has been dried in a convection oven with an accuracy of 0.5 %. Samples were crushed, massed (3.00 g), placed in tared crucibles in an oven at 100 °C for 2 h, cooled in a desiccator, and reweighed. Moisture was determined by mass difference pre- and post-drying. Moisture analysis results from the gravimetric method and the moisture analyzer had a standard deviation of ±0.3 %.

#### 2.5.2 Lipid content

Oil content from the potato chip samples was determined using a Soxhlet crude fat extraction with 2.00 g of ground potato chips, placed in a cellulose thimble, and subjected to refluxing petroleum ether solvent (100 mL), AOAC method #963.15 [50].

#### 2.5.3 Reducing sugars analysis

The quantity of reducing sugars was determined spectrophotometrically using Megazyme d-Glucose and d-Fructose assay kits (Neogen Corporation, Lansing, MI, USA) [51]. All potatoes, raw, control, and PEF treated, were flash frozen in liquid nitrogen, followed by homogenization in a Waring commercial blender (CB15, Stamford, CT, USA). 5.00 g of homogenate (or 0.5 g of crushed fried chips) was weighed into a 50 mL falcon tube, to which was added 10 mL of water. The suspension was shaken in a horizontal shaker for 1 h before centrifugation. The supernatant (1.00 mL) was placed in a different vial, with 1.00 mL of phenol: chloroform: isoamyl alcohol (25:24:1) standard for 20 s before centrifugation and collection of the supernatant. The solvent-extracted sugars were analyzed in triplicate in a 48-well microplate using a BioTek Epoch 2 Microplate Reader and Gen5 software (BioTek Epoch 2, BioTek Instruments, Inc., Winooski, VT, USA). Absorbance was taken at 340 nm to measure the amount of glucose and fructose present, respectively. Reducing sugars content was calculated by summing the amount of glucose and fructose.

#### 2.5.4 Asparagine quantification

Asparagine content was determined spectrophotometrically using a Megazyme l-Asparagine/l-Glutamine/Ammonia assay kit (Neogen Corporation, Lansing, MI, USA). One gram of the previous homogenate (or 0.1 g of fried chips) was weighed into a 15 mL falcon tube, to which 10 mL of 33 % ethanol (v/v) was added. The suspension was extracted using a horizontal shaker for 1 h before centrifugation. One mL of supernatant was used in 3 wells for analysis in triplicate using a 48-well microplate set to measure changes in absorbance at 340 nm indicative of asparagine quantity.

#### 2.5.5 Acrylamide quantification

Fried potato chip samples were placed in liquid nitrogen followed by grinding in a Waring commercial blender (CB15, Stamford, CT, USA) and uniformly pulverized. Homogenized samples (1.00 ± 0.02 g) were weighed and placed in a 50 mL centrifuge tube and spiked with 500 μL of ^13^C_3_ acrylamide (1,2,3–^13^C_3_, 99 %, Cambridge Isotopes Laboratories). The determination of acrylamide was carried out using dispersive solid phase extraction (QuEChERS, Quick, Easy, Cheap, Effective, Rugged, and Safe) [52] with hexanes (5 mL), nanopore water (10 mL), and acetonitrile (10 mL). Samples were 0.45 nm filtered and placed into 2 mL amber autosampler vials for Liquid Chromatography - Triple Quadrupole Mass Spectrometer (LC-TQMS). The LC system was an Agilent 1290 Infinity II system whereas the TQMS detector was an Agilent 6470B. Five microliters of sample were injected onto a Hypercarb column (100 mm × 3 mm, 3 μL, Thermo Scientific, Waltham, USA) at 60 °C. The mobile phase consisted of 0.1 % formic acid in water and 0.1 % formic acid in methanol with a flow rate of 0.3 mL/min. The following transitions were used for quantification purposes: quantification ion 72.1 → 55, (declustering potential 45 V, collision energy 12 V, cell accelerator voltage 4 V), confirmation ion 72.1 → 27, (declustering potential 45 V, collision energy 28 V, cell accelerator voltage 4 V), internal standard ion 75.1 → 58, (declustering potential 45 V, collision energy 12 V, cell accelerator voltage 4 V), and internal standard confirmation ion 75.1 → 30, (declustering potential 45 V, collision energy 32 V, cell accelerator voltage 4 V).

#### 2.5.6 Color quantification

The CIE L*, a*, and b* color of fried samples was measured using a colorimeter (Aeros D25 NC, Hunterlab, Reston, VA, USA) based upon tri-stimulus CIE color combination values, L* (lightness (100) to darkness (0)), a* (red (+) to green (−)), and b* (blue (+) to yellow (−)). Due to the uneven surface of the fried potato slices, a sample handling package for chips (L02-1014-741, Hunterlab, Reston, VA, USA) was used to provide a uniform sample presentation and repeatable results. One hundred and 50 g of fried chips were placed onto a sample dish attached to a compression ring, and using a compression pan, the chips were pressed firmly. After removing the compression pan and ring, the batch had a flat surface, which was uniform in height and opacity. A total of five color measurements were taken for each fried sample, using the Aeros D25 NC which averages five measurements per second and 25 measurements per rotational cycle of the turntable.

#### 2.5.7 Textural measurements

Compression tests were achieved by using a TA. XT Plus texturometer (Hamilton, USA) equipped with a multi-puncture stainless steel probe. Approximately 25.0 g of chips were loaded into an acrylic testing container. The TA. XT Plus settings were compression, 4 mm/s Test Speed, 10.0 Post-test speed, 60.0 mm distance, stop plot at the target position, probe height was calibrated and set to 65 mm before each test, which leaves a fixed 5.00 mm gap at the bottom of each stroke. Force vs distance curves were obtained and the results were expressed as 1) firmness, calculated using maximum forces; 2) crispness, calculated as the amount of small fractures; and 3) crunchiness calculated using linear distance (the length of a line joining all fracture points in the force-deformation curve) between the first and the last puncture peaks registered.

### 2.6 Statistical analysis

Each experimental condition consisted of 3 replicates, each comprising 5 frying batches. The statistical significance between treatments was calculated using analysis of variance (ANOVA) with a significance level of 95 %, followed by pairwise comparisons with adjustment for multiple comparisons. Multiple comparisons, or ‘post-hoc tests’ were applied to observe differences between experimental groups and to clarify the differences between particular pairs of groups; particularly we used the Bonferroni method. All statistical analysis was performed using IBM SPSS Statistics version 29 (IBM Corporation, New York, NY, USA).

## 3 Results and discussion

### 3.1 Moisture and crude fat content

Raw potatoes had an initial moisture content of 76.06 %, which was reduced to 0.73–2.35 % moisture by frying. The final moisture content in the fried chips for Low-PEF, High-PEF, and untreated chips was 1.61 %, 1.52 %, and 1.12 % respectively (Table 4). PEF treatment of potatoes can enhance water diffusion [49], which promotes faster frying efficiency. We were able to shorten frying times (from 180 s to 150 s) by 16 % when frying at 170 °C and by 10 % (from 150 s to 135 s) when frying at 185 °C with PEF-treated Lamoka potatoes, reaching the desired moisture content of <2.5 % in the potato chips 30 s sooner at 170 °C and 15 s sooner at 185 °C than with the untreated potatoes (Table 4).

**Table 4:** Moisture and crude fat content of untreated and PEF-treated potato chips. Moisture is expressed in percentage and is compared between the raw potato versus the final potato chips. The crude fat is expressed as a percentage and the reduction value compares the PEF-treated versus the control of the same frying parameters.

	Frying parameters		Raw potatoes	Potato chips		
	Temperature (°C)	Time (s)	Moisture (%)	Moisture (%)	Crude fat (%)	Reduction (%)
Control	170	150	75.19 ± 0.81	1.57 ± 0.16^a^	43.96 ± 0.64^ab^	–
Low-PEF			75.87 ± 1.33	1.68 ± 0.16 ^b^	40.90 ± 0.50^ac^	7.0
High-PEF			75.90 ± 0.94	2.35 ± 0.13 ^ab^	42.76 ± 0.34^bc^	2.7
Control	170	180	76.22 ± 1.09	1.16 ± 0.38	44.83 ± 0.47^de^	–
Low-PEF			76.87 ± 0.08	1.35 ± 0.09	42.18 ± 0.34^df^	5.9
High-PEF			79.69 ± 1.44	1.30 ± 0.16	43.43 ± 0.32^ef^	3.1
Control	185	135	75.96 ± 1.07	1.02 ± 0.15 ^cd^	47.04 ± 0.50^gh^	–
Low-PEF			76.08 ± 0.56	1.58 ± 0.15^c^	44.48 ± 0.68^g^	5.4
High-PEF			81.98 ± 1.31	1.58 ± 0.15 ^d^	43.33 ± 0.43^h^	7.9
Control	185	150	71.60 ± 1.46	0.73 ± 0.08^f^	48.05 ± 0.76^j^	–
Low-PEF			80.67 ± 0.39	1.84 ± 0.27^f^	44.42 ± 0.29^jk^	7.5
High-PEF			73.18 ± 0.04	0.86 ± 0.20	45.39 ± 0.46^k^	5.5

Different letters in the same columns indicate significant differences among samples according to Bonferroni multiple pairwise comparisons (p < 0.05).

PEF treatment of Lamoka potatoes resulted in a reduction of oil uptake by the chips during frying, compared to the control fried sample. At 170 °C, we observed the greatest reduction with the Low-PEF parameter, averaging 6.45 % compared to the control whilst, at 185 °C the High-PEF reduced by an average of 6.7 %. These results suggest that absorption of oil by PEF-treated samples does not depend only on the initial moisture content, or the frying conditions, but also on the induced tissue electroporation phenomenon. In a very similar manner, Fauster et al. (2018) observed that PEF-treatment of potatoes led to a reduction from 6.8 to 7.5 % in the oil content of the final French fries. Ignat et al. (2015) attributed this result to the transfer of water and intracellular substances to the surface of the fries due to the PEF-induced electroporation; the higher amount of accessible water at the surface creates a mass transfer barrier and reduces oil uptake. Liu et al. (2022) and Janositz et al. (2011) reported that oil uptake from PEF-treated potato slices was between 34 and 39 % lower compared to untreated samples. Zhang et al. [53] observed that increasing the pulse number from 500 to 8000 pulses resulted in a decrease in oil content by 12.2 %; increasing pulse width from 5 μs to 40 μs led to a rise in oil content of 5.4 %, and electric field strength increase from 1 kV/cm to 10 kV/cm decreased oil content by 21.2 %. Our findings were a reduction of oil uptake of 6.45 % for Low-PEF and 4.8 % for High-PEF, this low oil reduction might be related to a continuous frying style which takes shorter frying times than batch frying.

Frying is mainly a drying process that involves heat and mass transfer. After initial heating of the potato slices through the surrounding oil, surface boiling begins including water vaporizing and the formation of bubbles. Moisture is transferred from the surface to the oil and later by diffusion of inner cellular liquid to the surface. The water vapor layer on the potato surface acts as a barrier against the oil and depends on the vapor pressure difference between the potato slice moisture and oil, which influence the rate of drying [54]. Due to the permeabilized cell membranes of PEF-treated tissue cell liquid diffusion from the core to the surface is enhanced, which results in a higher-pressure difference and thus a thicker water vapor layer, reducing dehydration and fat uptake.

The observed oil uptake reduction is mostly a benefit to the end consumer and an advantage to the snacking industry providing a healthier (less fat in the chips) version of this well-liked potato product when adopting the PEF technology.

### 3.2 Maillard reaction substrates

Reducing sugars and asparagine are the precursors for acrylamide formation in potato products [55]. Table 5 shows the glucose, fructose, and free asparagine content (mg/100 g of tuber) in raw Lamoka potatoes and slices subjected to a 1-min rinsing and dewatering step, described as control, or PEF pre-treatment before slicing, 1-min rinsing, and dewatering by pat-drying the potato slices with paper towels.

**Table 5:** Maillard substrates (glucose, fructose, and asparagine) content in raw potatoes, control, and PEF treatments (after slicing, rinsing, and dewatering). Results are expressed in mg/100 g of potato, mean values, and percentage of Relative Standard Deviation (%RSD) from n = 12. Reduction percentages are calculated in relation to the raw potato sample.

Sample	Glucose			Fructose			Asparagine		
	Mean	% RSD	Reduction (%)	Mean	% RSD	Reduction (%)	Mean	% RSD	Reduction (%)
Raw	120.2	5.6	–	60.9	13.4	–	456.5	21.8	–
Control	112.9	6.1	6.1	58.6	14.2	3.8	411.6	5.1	9.8
Low-PEF	87.4	5.0	27.4	51.4	8.3	15.5	263.1	12.5	42.4
High-PEF	83.1	6.2	30.9	49.0	12.8	19.5	248.0	12.0	45.7

The reduction in reducing sugars and asparagine was significantly different between the control and both PEF treatments. The glucose levels are observed to drop from their highest levels in raw potato (120.2 mg/100 g) to the lowest value in the High-PEF treatment of 83.1 mg/100 g. Fructose levels didn't have such a dramatic drop, the initial value in the raw potatoes was 60.9 mg/100 g, which was later reduced to 49.0 mg/100 g for the High-PEF treatment. Asparagine was the most impacted by the PEF treatment, with an initial content of 456.5 mg/100 g dropping to 248 mg/100 g for the High-PEF treatments.

It is well established that acrylamide precursors, reducing sugars and amino acids, are leached out by rinsing and blanching [56]. PEF treatment has been suggested as an alternative to blanching to reduce acrylamide precursors before frying [17]. The reduction in acrylamide precursors is attributed to PEF induced resulting in leakage of water-soluble molecules from potato tissue cells into water during the rinsing step. We observed that PEF treatment promoted a significant reduction in glucose, fructose, and free asparagine, consistent with the literature [54]; this reduction has a direct impact on the acrylamide formed during Maillard reactions that occur during frying therefore suggesting a health benefit on the consumers of PEF-treated chips when these precursors have reduced significantly.

### 3.3 Acrylamide

**Fig. 2:**
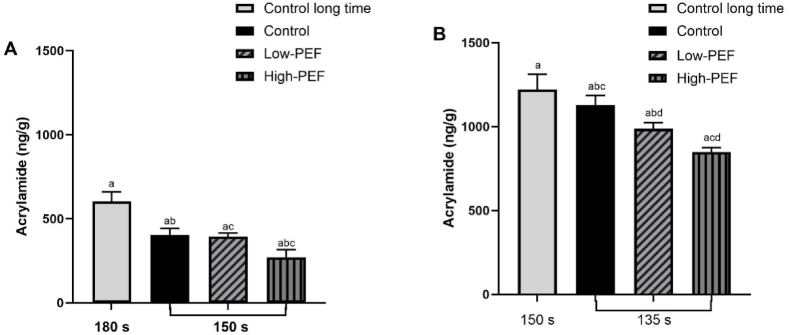
Acrylamide levels in potato chips fried at 170 °C (A) and 185 °C (B). Results are expressed as means ± standard deviations (error bars) of n = 36. The means with same letters are significantly different (p < 0.05).

We quantified acrylamide in Lamoka potato chips across 2 temperatures, 2 frying times, and 2 PEF treatment levels (Table 3). The results were acrylamide levels that ranged in concentration from 228 ng/g for High-PEF frying at 170 °C for 150 s to 1288 ng/g for control frying at 185 °C for 150 s. Fig. 2A shows the comparison between untreated and PEF-treated potato chips fried at 170 °C, for 150 s or 180 s. Fig. 2B shows the comparison between untreated and PEF-treated potato chips fried at 185 °C, for 135 s or 150 s. The acrylamide levels showed dependence on the frying temperature, the time of frying (135, 150, or 180 s), and the PEF treatment level (0.75 kJ/kg or 1.5 kJ/kg). Acrylamide levels are higher for chips fried at higher temperatures for a longer time. For control frying at 170 °C for 180 s, the quantified acrylamide value is 604 ng/g whereas at the same temperature frying for 150 s, the control had 405 ng/g. PEF had a positive effect on the quantified acrylamide at the same frying temperature for 150 s, with values of 394 ng/g for Low-PEF and 272 for High-PEF. Similarly, for the frying temperature of 185 °C, the control fried for 150 s accounted for 1221 ng/g, while the control fried for 135 s had 1130 ng/g. PEF also had a positive effect on acrylamide values at his temperature reducing to 988 ng/g for Low-PEF and 849 ng/g for High-PEF. The biggest reduction observed in both frying temperatures was with the High-PEF parameter (1.5 kJ/kg) reducing 32.84 % compared to the control at 170 °C, followed by the High-PEF reducing 24.87 % compared to the control at 185 °C.

Acrylamide formation decreases when PEF treatments are applied, and the High-PEF treatment demonstrated a consistently higher decrease in acrylamide. These findings are similar to those reported by Genovese et al., 2019 in which the acrylamide content of potato crisps produced by PEF-treated potatoes was about 30 % lower than those for untreated chips. PEF may considerably reduce, through diffusion, the precursor content available to participate in the Maillard reaction, and hence, the amount of acrylamide formed during frying is reduced [57].

We observed a direct correlation between the acrylamide formed during the frying of potato chips and the concentration of the precursors. Free asparagine in the control Lamoka potatoes was 411.6 mg/100 g, and reducing sugars were 173.5 mg/100 g, yielding acrylamide levels of 604 ng/g when fried at 170 °C for 180 s and 1221.3 ng/g when fried at 185 °C for 150 s. On the same relation, we observed the lowest acrylamide levels in the potato chips when the precursors were lower than the control; free asparagine in the High-PEF Lamoka potatoes was 248 mg/100 g, the reducing sugars were 145 mg/100 g, yielding acrylamide levels of 272 ng/g when fried at 170 °C for 150 s and 849 ng/g when fried at 185 °C for 135 s in the potato chips.

The optimal conditions for PEF and continuous frying of Lamoka potato chips were found to be a frying temperature of 170 °C for 150 s with a PEF treatment of 1.5 kJ/kg. Our results obtaining a 32.84 % reduction of acrylamide are consistent with other studies [58,59] and support the practice of frying at lower temperatures to mitigate acrylamide formation in fried potato chips. Frying at 185 °C for 135 s (Fig. 2B) led to an acrylamide content of 1221.3 ng/g, this value is greater than 604 ng/g formed when frying at 170 °C for 180 s (Fig. 2A) in Lamoka chips.

According to California Proposition 65, where potato chip producers are required to add a warning label to their products if the acrylamide value exceeds 275 ng/g; our process of frying potato slices at 170 °C for 150 s with High-PEF yielded potato chips with acrylamide content of 272 ng/g; which speaks to the health implications for consumers and the quality and safety of these chips.

### 3.4 Color

The effect of PEF treatment is that chips have a uniform, light color and less brown (a combination of a high value of a* and b*) after frying when PEF is used. The best outcomes in terms of redness (a*) and yellowness (b*) combinations yielding in pale yellow chips were both PEF treatments fried at 170 °C for 150 s as well as High-PEF fried at 185 °C for 135 s, as shown in Table 6.

**Table 6:** The effect of PEF treatment on the L*, a*, and b* values of fried potato slices at different frying conditions.

Frying conditions	Color parameters	Control	Low-PEF	High-PEF
170 °C for 150 s	L* a* b*	66.14 ± 0.07^a^ 3.30 ± 0.02 ^ab^ 31.7 ± 0.95	71.17 ± 0.16 ^b^ 2.45 ± 0.02 ^bc^ 31.31 ± 0.04	68.16 ± 0.05 ^ab^ 2.75 ± 0.01 ^ac^ 31.87 ± 0.06
170 °C for 180 s	L* a* b*	63.10 ± 0.05^a^ 7.54 ± 0.03 ^ab^ 35.78 ± 0.05^a^	63.21 ± 0.44 ^b^ 6.08 ± 0.02 ^bc^ 35.63 ± 0.05 ^b^	61.20 ± 0.10 ^ab^ 6.29 ± 0.05 ^ac^ 34.36 ± 0.04 ^ab^
185 °C for 135 s	L* a* b*	67.76 ± 0.03 ^ab^ 3.07 ± 0.04^a^ 32.10 ± 0.04 ^ab^	70.58 ± 0.28 ^bc^ 5.56 ± 0.02 ^ab^ 31.90 ± 0.03 ^bc^	72.47 ± 0.08 ^ac^ 2.73 ± 0.06 ^b^ 31.37 ± 0.13 ^ac^
185 °C for 150 s	L* a* b*	65.95 ± 0.08 ^ab^ 6.06 ± 0.02^a^ 36.99 ± 0.04	65.18 ± 0.08 ^bc^ 5.46 ± 0.02 ^b^ 36.00 ± 0.04	58.38 ± 2.14 ^ac^ 8.45 ± 1.20 ^ab^ 36.24 ± 1.57

The data is presented as mean ± standard deviation (n = 25). The means in the same row for each treatment sharing the same lowercase letter in superscripts are significantly different at *p* < 0.05 based on ANOVA and Bonferroni multiple pairwise comparison tests.

Color attributes that are perceived by consumers may influence consumers’ product acceptance. Our results with PEF yielded uniform color chips that might not even be perceived by consumers because the colorimetric assessments performed show very minimal differences, which is beneficial to the potato chip producers; color quality is not been impacted when incorporating a PEF technology.

Our colorimetric results are shown in Table 6; the lightness of the potato chips decreases when frying for longer times. At 170 °C, when frying at 150 s, when comparing the PEF treatments vs, the control, we observed lighter color chips by 7.1 % for Low-PEF and by 3.0 % for High-PEF at the same condition. At 185 °C, when frying for 135 s, PEF treatments increase the lightness of the fried chips by 4.0 % for Low-PEF and by 6.5 % for High-PEF vs control.

Yellowness (b*) is the chromatic parameter corresponding to the yellow color when it is positive (+) and the blue color when it is negative (−); in our results, yellowness had a bigger dependence on the frying time than on the PEF treatment. In both cases, frying at 170 °C and 185 °C results in smaller yellow values when frying for short times with or without PEF treatments. Redness (a*) is the chromatic parameter corresponding to the red color when it is positive (+) and the green color when it is negative (−). Similarly, frying at 170 °C and 185 °C results in smaller red values when frying for short times. PEF affected reducing the redness of the samples (3.30 for control, vs. 2.45 for Low-PEF and 2.75 for High-PEF at 170 °C and 7.54 for control vs. 6.08 for Low-PEF and 6.29 for High-PEF at 185 °C).

Genovese et al. (2019) and Ignat et al. (2015) observed similar results in potato chips that were light in color and uniform pale yellow after frying when using PEF pretreatments.

### 3.5 Textural analysis

**Fig. 3:**
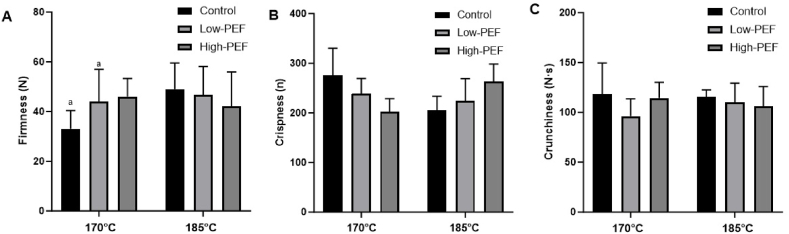
Firmness (A), crispness (B), and crunchiness (C) of untreated, Low-PEF, and High-PEF potato chips at different frying temperatures. Results are expressed as means ± standard deviations (error bars) of n = 36. The means with the same letters are significantly different (p < 0.05).

As shown in Fig. 3A, we observe at 170 °C that firmness is higher between the PEF treatments (Low-PEF, 44 N and High-PEF 46 N) and the control (33 N), this parameter is only significant between the control and the PEF treatment at 0.75 kJ/kg. At 185 °C, firmness is higher compared to the lower frying temperature for the control and Low-PEF, and the firmness reduces from control (49 N) to Low-PEF (46.67 N) and High-PEF (42.17 N). Firmness is very similar across chips regardless of PEF treatment or frying temperature demonstrating that PEF treatments do not have a textural impact on the final potato chip.

As shown in Fig. 3B, crispness at 170 °C decreases from control (276 peaks) to Low-PEF (239 peaks) and some more to High-PEF (203 peaks). The opposite trend is seen at 185 °C, increasing from control (205 peaks) to Low-PEF (224 peaks) and High-PEF (263 peaks). Similarly, to firmness, crispness values are very alike across chips regardless of PEF treatment or frying temperature demonstrating that PEF treatments do not have an impact on the crispness of the potato chip. Dry crisp products are those that contain cells or cavities filled with air instead of water. Crispness describes a quick fracture under small strain stresses owing to low water content in the surface layer of the potato slice [60]. It is important to note that the effect of PEF treatment on fried foods cannot be assumed since crispness is highly dependent on frying parameters, oil content, moisture content, porosity, and roughness of the surface [61].

At 170 °C, the control had the highest value (118.09 N∙s) followed by High-PEF (114.53 N∙s) and decreasing to Low-PEF decreasing (96.17 N∙s). Compared to 185 °C, the values are alike, slightly decreasing from control (115.33 N∙s) to Low-PEF (110 N∙s) and finally to High-PEF (106.22 N∙s). Force-deformation curves of crunchy dry foods lose their jaggedness when moisture is added [62]. Fig. 3C demonstrates that crunchiness values were very similar, not losing jaggedness in a significant matter, regardless of frying temperature or PEF treatment. Crunchiness values are extremely alike across chips regardless of PEF treatment or frying temperature demonstrating that PEF treatments do not have an impact on this textural attribute of the potato chip. Cahayadi et al. [63] reported that fried chips produced from PEF-treated potatoes were perceived to be crunchier compared to potato chips from untreated potatoes. Our findings showed no significant differences in the crispness or crunchiness of chips when comparing untreated control versus PEF-treated ones. Texture attributes might be perceived by consumers and influence consumers’ product acceptance. Our results with PEF yielded no textural differences on the chips, no changes would be perceived by consumers, which is beneficial to the potato chip producers when incorporating a PEF technology.

The findings in this study have been seen in light of some potential limitations. The first limitation is the frying trials are limited to a small batch size that does not represent an industrial potato chip operation or a multi-zone fryer behavior. The second limitation concern is the color and texture results are quantitatively significant but have not been tested by a sensory panel.

## 4 Conclusion

Potato chips in the United States are commonly made with Lamoka potatoes. In this study we measured the effect PEF technology had on three factors: 1) Moisture and fat content of potato chips, 2) Acrylamide formation and its precursors, and 3) The organoleptic quality of the chips. PEF was shown to shorten frying times by 10 % compared to conventional processing. An estimated 1165 kWh is needed to fry 2500 kg of finished potato chips per hour with a yearly energy cost of $204,108 [64]; when using a PEF system, the frying time is shortened by 10 % with an energy usage is 1048 kWh for the same amount of finished product yielding a yearly energy cost of $183,610; a $20,500 difference. The fat content of the potato chips was reduced by 6.45 % when comparing Low-PEF vs. control. This reduction has an impact on the health of potato chip consumers, as well as the snack industry creating consistent lower-fat potato chips when incorporating PEF technology as part of their existing processing lines. Both PEF treatments had a significant effect on acrylamide-forming precursor reduction; however, the highest impact was with the 1.5 kJ/kg where glucose was reduced by 30.9 %, fructose by 19.5 %, and asparagine by 45.7 % compared to the control. This precursor reduction diminished the acrylamide content by 32.83 % when frying at 170 °C and by 24.86 % at 185 °C. Acrylamide formation is dependent on the frying temperature, doubling its value when frying at 170 °C for 180 s (604 ng/g) and 185 °C for 150 s (1221 ng/g). The implications of adopting PEF technology to decrease the amount of acrylamide in potato chips have significant positive results, demonstrating that utilizing this technology yields potato chips that can be sold to consumers without any warning labels for acrylamide safety concerns as per California Proposition 65. In the final potato chips, we observed lighter color products when using a PEF pretreatment before frying, and no significant changes were observed in the textural properties analyzed like firmness, crispness, and crunchiness. This study advances the understanding of quantitative benefits when adopting a PEF pretreatment using Lamoka potatoes on continuous-style frying. PEF treatments showed positive effects when shortening frying times and using a lower frying temperature with positive impacts on moisture, oil uptake, acrylamide, and quality aspects of the potato chips. The adoption of PEF technology is quite easy, by incorporating a belt system with continuous PEF pretreatment into a current processing line. The benefits of adopting this technology do not only include smoother cuts, less wear-and-tear on slicer blades, the possibility for newer cuts, and energy savings but also obtaining consistent lower-fat, lighter color, and safer potato chips in terms of acrylamide content without sacrificing textural properties, known by customers.

## Data availability statement

The data supporting this study's findings are available from the corresponding author, upon request.

## Funding sources

We acknowledge support from the Institutional Development Awards (IDeA) from the National Institute of General Medical Sciences of the National Institutes of Health under Grants #P20GM103408, P20GM109095, and 1C06RR020533. We also acknowledge support from BSU- Biomolecular Research Center, RRID:SCR_019174, supported by the National Science Foundation, Grants #0619793 and #0923535; the M. J. Murdock Charitable Trust, and the Idaho State Board of Education.

## CRediT authorship contribution statement

**Priscila Santiago-Mora:** Writing – original draft, Visualization, Validation, Project administration, Methodology, Investigation, Formal analysis. **Mark Skinner:** Validation, Investigation. **Alyssa Hendricks:** Investigation. **Tauras Rimkus:** Investigation. **Brian Meyer:** Supervision, Methodology, Conceptualization. **Jim Gratzek:** Methodology, Conceptualization. **Shin Pu:** Investigation. **Luke Woodbury:** Investigation. **Laura Bond:** Formal analysis. **Owen McDougal:** Writing – review & editing, Validation, Supervision, Project administration, Funding acquisition, Conceptualization.

## Declaration of competing interest

The authors declare that the work described in this article has not been published previously, nor is it under consideration for publication in any other venue. All authors have approved the content of this manuscript for publication. The authors have no declarations of interest to report, i.e., Declarations of interest: none.

## Abbreviations

PEF: Pulsed Electric Field
Asn: Asparagine
*E*: electric strength
*f*: pulse frequency
*N*: pulse number
*W*: specific energy input
τ: pulse width
*t*: pulse duration
QuEChERS: Quick, Easy, Cheap, Effective, Rugged, and Safe
LC-TQMS: Liquid Chromatography - Triple Quadrupole Mass Spectrometer
L*: Lightness
a*: Yellowness

b*: Redness
